# Reducing inappropriate antibiotic prescribing for acute uncomplicated bronchitis: a systemwide quality improvement project

**DOI:** 10.1017/ash.2024.465

**Published:** 2024-12-20

**Authors:** Sabrina Williams, Julie Engels, Sara Ogrin, Nolan Rossman, Rosemary Olivero

**Affiliations:** 1 Michigan State University College of Human Medicine, Grand Rapids, MI, USA; 2 Helen DeVos Children’s Hospital of Corewell Health, Grand Rapids, MI, USA

## Abstract

This systemwide quality improvement project examined whether a bundle of antimicrobial stewardship interventions reduced the proportion of inappropriate antibiotic prescriptions in ambulatory encounters for adults with acute uncomplicated bronchitis. There was an overall reduction in the proportion of inappropriate prescriptions from pre- to postinterventions (44.9%–32.5% [*P* < 0.001]).

## Introduction

Antimicrobial resistance has been identified by the Centers for Disease Control and Prevention (CDC) as well as the World Health Organization as one of the leading threats to human health.^1,2^ Overprescribing of antimicrobials is estimated to contribute to the development of 2.8 million cases of antimicrobial-resistant infections in the United States every year.^1^ Antibiotic prescribing is common in ambulatory visits for acute respiratory tract infections (ARTIs),^3^ and it is estimated that approximately 50% of antibiotics prescribed for these encounters are unnecessary.^4^ Antimicrobial stewardship programs (ASPs) have been developed in inpatient and ambulatory settings to reduce inappropriate antibiotic prescriptions (IAPs),^5^ and ambulatory ASPs became Joint Commission-mandated in most ambulatory settings in 2019.^6^ Ambulatory ASPs have been shown to decrease IAPs for select ARTIs.^7^ However, there remains a great need to optimize and expand ASPs and their initiatives to further reduce IAPs.

One ARTI for which antimicrobials are frequently prescribed is acute uncomplicated bronchitis (AUB).^8,9^ The most recent evidence-based guideline for the treatment of AUB includes the key recommendation to “avoid prescribing antibiotics for AUB.” According to this guideline, antibiotics should be reserved for treatment AUB only if chronic lung conditions or concurrent diagnoses of other bacterial conditions requiring antibiotic treatment are diagnosed.^10^

The aim of this study was to assess if a bundle of antimicrobial stewardship interventions (ASIs) in a large healthcare system impacted the proportion of IAPs for AUB in adults in ambulatory care visits.

## Methods

This was a quasi-experiment quality improvement (QI) study comparing the proportion of IAPs pre- versus postintervention in a health system’s ambulatory sites over a 2-year period. Institutional Review Board approval was sought and was waived as the study was identified as QI (nonhuman) research. A bundle of ASIs for AUB began in January 2021 (Table 1): (1) retrospective auditing of IAPs for AUB; (2) quarterly reporting of department-, clinic- and provider-level IAPs for AUB; (3) educational webinars on ASP and evidence-based guidelines for treatment of AUB; and (4) best practice alerts in the electronic medical record (EMR) when antimicrobials were prescribed for AUB. The preintervention period was January 1, 2020, through December 31, 2020, and the postintervention period was January 1, 2021, through December 31, 2021.

**Table 1.** Summary of antimicrobial stewardship interventions during the pre- and postintervention periods

Intervention	Timeline	Description	Comments
Auditing	January 2021–December 2021	Retrospective auditing of inappropriate prescribing for acute uncomplicated bronchitis	Automated electronic medical record report with patient- and department-level data used to assess the appropriateness of antimicrobial prescribing for acute uncomplicated bronchitis.
Quarterly reporting	Report dates: 1) March 2021 2) June 2021 3) September 2021 4) December 2021	Antibiotic prescribing reports finalized and sent to all ambulatory practices; included department-, clinic- and provider-level inappropriate antimicrobial prescribing for acute uncomplicated bronchitis	Reports sent to each ambulatory site lead and practice manager or supervisor.
Educational webinar	August–September 2020	Topic: Introduction to ambulatory stewardship	All ambulatory providers and practice managers invited to attend; webinars were broadcast in real time twice in 1 week, with recordings made available. Content: Overview of antimicrobial stewardship, global burden of antimicrobial resistance, review of ambulatory stewardship process and methodology.
Educational webinar	December 2020	Topic: Management of acute uncomplicated bronchitis	All ambulatory providers and practice managers invited to attend; webinars were broadcast in real time twice in 1 week, with recordings made available. Content: Review of evidence-based guidelines on the diagnosis, evaluation treatment of acute bronchitis, and both pharmacologic and nonpharmacologic management of the condition, review of ambulatory stewardship process and methodology.
Best practice alerts active in electronic medical record	January 2021–December 2021	Real-time alert in electronic medical record appeared if antimicrobials were prescribed in an encounter where a bronchitis diagnosis was entered by the provider	Content of alert: “This patient was prescribed an antibiotic with a diagnosis of bronchitis, a viral infection in the majority. Antibiotics are not generally indicated. The Centers for Disease Control and Prevention has recommended avoiding antibiotics unless the patient also has a chronic lung disease, immune deficiency, or an alternate bacterial infection.” A prompt to remove the antibiotic order(s) appears, prepopulated to remove the antibiotic orders. If the prompt to remove the antibiotic is changed to continue the antibiotic, an acknowledged reason must be completed, and choices include “Alternate bacterial infection,” “Comorbidity,” and “Other-see comments.” A prompt prepopulated with “Remove” the antibiotic order.

Weekly automated reports were accessed from a reporting platform in the institution’s EMR (Epic Hyperspace®). Ambulatory patient encounters were identified using the 10^th^ revision of the International Statistical Classification of Diseases (ICD-10) codes J20.9 and J20.8 for “bronchitis.” Ambulatory encounters included visits to urgent care as well as primary care sites (internal medicine, internal medicine-pediatrics, and family medicine) and included both in-person and virtual visits. Encounters for individuals less than 18 years of age, duplicate encounters, and follow-up visits for the same instance of illness were excluded.

Encounters were coded as “appropriate” if antibiotics were prescribed with the diagnosis of AUB and only if specific underlying conditions were documented in the patient’s EMR (chronic obstructive pulmonary disease, emphysema, pulmonary fibrosis, bronchiectasis, or immunodeficiency), or another diagnosis was made that required antimicrobial treatment (eg, sinusitis, community-acquired pneumonia), or no antimicrobial was prescribed with the diagnosis of AUB. Encounters were coded as “inappropriate” if an antibiotic was prescribed without an alternate diagnosis requiring antimicrobial treatment or if none of the aforementioned comorbid conditions were documented in the encounter, patient history, or patient problem list.

The analysis for this study included descriptive and inferential statistics for both patient demographics and monthly comparisons of the proportions of IAPs between groups. All numeric variables were nonnormal between groups, displayed as median (25^th^, 75^th^ percentile), and tested with the Wilcoxon rank sum test. Categorical variables (facility type, sex, and ethnicity) were displayed as count (percentage) and tested via χ^2^ analysis. Demographic comparisons were assessed at an alpha of 0.05, while a Bonferroni adjustment was applied to the *P*-values for the monthly comparison to correct for multiple testing.

## Results

**Figure 1.**
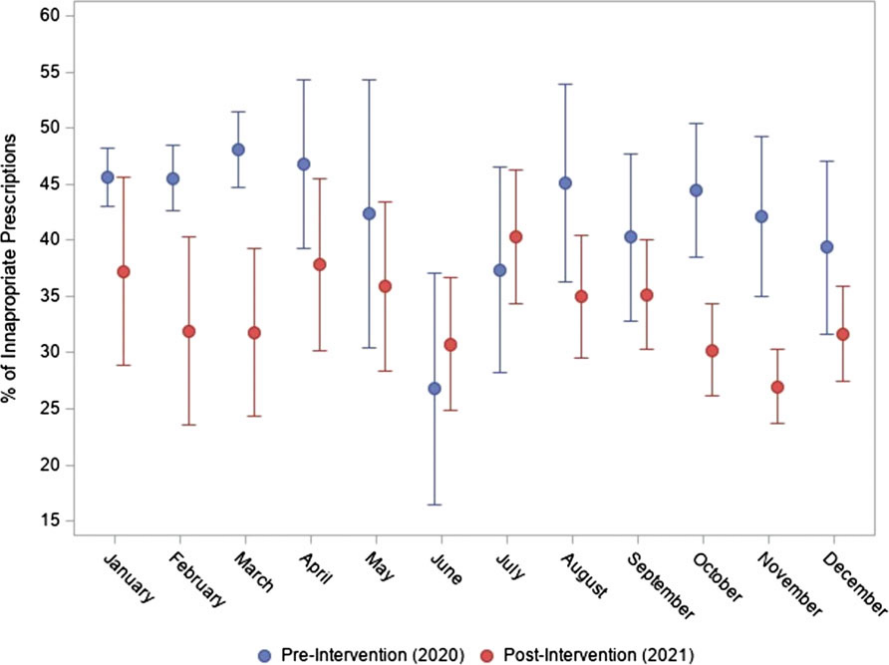
Monthly comparisons of inappropriate prescriptions by group.

A total of 8,176 encounters were included in this analysis. There were 4,694 encounters in the preintervention period and 3,482 encounters in the postintervention period (Figure 1). There was an overall decrease in IAPs for AUB preintervention compared to postintervention (44.9% vs 32.5%, [*P* < .001]). Additionally, there was a decrease in IAPs for AUB from preintervention to postintervention in the following months: March (48.1%–31.8%, *P* = .0002), October (42.2%–27.0%, *P* < .0001), and November (42.2%–27.0%, *P* < .0001); declines in IAPs were not statistically significant when comparing pre- to postintervention prescribing in the other months. Of note, there was an overall increase in urgent care visits from 32.6% in 2020 to 37.9% in 2021, while clinic visits decreased from 67.4% to 62.1% in the same time frame. There was an association between IAPs and facility type, with an overall higher proportion of IAPs in clinics compared to urgent care sites (41.3% vs 36.5%, *P* < .0001). There were no differences in IAP rates for AUB among demographic groups, including race, gender, or primary language in the preintervention or postintervention periods.

## Discussion

This study corroborates previous reports of high rates of antimicrobial use for AUB in ambulatory care,^8,9^ despite long-standing recommendations to avoid antibiotic use in AUB for most patients diagnosed as having this condition. The ASIs demonstrated a significant decline in IAPs for AUB, with salient decreases in IAPs during 3 months of the typical peak respiratory viral season in Michigan; these declines are likely due to the increased number of acute care visits for bronchitis during these months, allowing for increased power to detect statistically significant changes.

Antimicrobial stewardship initiatives are institution- and/or health system-dependent and can vary significantly in their structure, duration, and impact. This study demonstrates that a bundle of ASIs can lead to a decrease in IAPs for AUB in adult patients. A previous study also demonstrated decreases in IAPs for ARTIs with a bundle of ASIs but also found that IAP rates rebounded after ASIs ceased.^7^ It is currently recommended by the CDC that institutions and healthcare systems engage in improving antibiotic prescribing by developing and implementing strategies that align with evidence-based recommendations for the diagnosis and management of infections. As inappropriate prescribing may vary by practice location, focusing ASIs toward sites with higher rates of IAP may be effective.

This study has several limitations. Data collection was retrospective in nature, and inclusion in the study was based on provider-selected ICD-10 codes for bronchitis, which is subject to selection bias. Inappropriate prescribing was based on whether specific underlying conditions were documented in the encounter notes, medical histories, and problem lists in the EMR, which may not be completely accurate for all patient encounters. Conversely, this study was strengthened by the data set size, which led to smaller margins of error and highly reliable results. Because the study included multiple ASIs in the bundle, it cannot be determined which intervention was the most impactful in reducing IAP for AUB. Lastly, the durability of the impact of the ASIs used in this study will be evaluated over time.

This study adds to the growing body of evidence supporting that ASIs can meaningfully decrease IAP for ARTIs. Further studies are needed in different healthcare settings to confirm these findings, as well as compare which of many possible ASIs are most effective at reducing inappropriate antibiotic use.
